# Assessing the Involvement of Selected Phenotypes of *Pseudomonas simiae* PICF7 in Olive Root Colonization and Biological Control of *Verticillium dahliae*

**DOI:** 10.3390/plants10020412

**Published:** 2021-02-23

**Authors:** Nuria Montes-Osuna, Carmen Gómez-Lama Cabanás, Antonio Valverde-Corredor, Roeland L. Berendsen, Pilar Prieto, Jesús Mercado-Blanco

**Affiliations:** 1 Departamento de Protección de Cultivos, Instituto de Agricultura Sostenible, Agencia Estatal Consejo Superior de Investigaciones Científicas (CSIC), Avenida Menéndez Pidal s/n, Campus “Alameda del Obispo”, 14004 Córdoba, Spain; nuriamontes@ias.csic.es (N.M.-O.); cgomezlama@ias.csic.es (C.G.-L.C.); valverde@ias.csic.es (A.V.-C.); 2 Plant–Microbe Interactions, Department of Biology, Faculty of Science, Utrecht University, Padualaan 8, 3584 CH Utrecht, The Netherlands; R.L.Berendsen@uu.nl; 3 Departamento de Mejora Genética Vegetal, Instituto de Agricultura Sostenible, Agencia Estatal Consejo Superior de Investigaciones Científicas (CSIC), Avenida Menéndez Pidal s/n, Campus “Alameda del Obispo”, 14004 Córdoba, Spain; pilar.prieto@ias.csic.es

**Keywords:** biocontrol, biofilm, confocal laser scanning microscopy (CLSM), copper tolerance, defoliating (D) pathotype, endophyte, integrated disease management, *Olea europaea* L., phytase activity, Verticillium wilt of olive

## Abstract

*Pseudomonas simiae* PICF7 is an indigenous inhabitant of the olive (*Olea europaea* L.) rhizosphere/root endosphere and an effective biocontrol agent against Verticillium wilt of olive (VWO), caused by the soil-borne fungus *Verticillium dahliae*. This study aimed to evaluate the potential involvement of selected phenotypes of strain PICF7 in root colonization ability and VWO biocontrol. Therefore, a random transposon-insertion mutant bank of *P. simiae* PICF7 was screened for the loss of phenotypes likely involved in rhizosphere/soil persistence (copper resistance), root colonization (biofilm formation) and plant growth promotion (phytase activity). Transposon insertions in genes putatively coding for the transcriptional regulator CusR or the chemotaxis protein CheV were found to affect copper resistance, whereas an insertion in *fleQ* gene putatively encoding a flagellar regulatory protein hampered the ability to form a biofilm. However, these mutants displayed the same antagonistic effect against *V. dahliae* as the parental strain. Remarkably, two mutants impaired in biofilm formation were never found inside olive roots, whereas their ability to colonize the root exterior and to control VWO remained unaffected. Endophytic colonization of olive roots was unaltered in mutants impaired in copper resistance and phytase production. Results demonstrated that the phenotypes studied were irrelevant for VWO biocontrol.

## 1. Introduction

Olive (*Olea europaea* L. subsp *europaea* var. *europaea*) is an important crop widely distributed across the Mediterranean Basin, the region where 98% of the world’s olive cultivating acreage is concentrated [1]. Moreover, this crop and its associated agri-food industry constitute one of the main economic drivers for the agricultural sector in many countries of this geographical area. Verticillium wilt of olive (VWO) caused by the soil-borne fungus *Verticillium dahliae* Kleb. is considered one of the most serious biotic constraints affecting this woody crop, a devastating disease which is extremely difficult to control (reviewed by [2]). The persistence of dormant structures (microsclerotia) of the pathogen in soils, its broad host range, its survival inside xylem vessels during its parasitic phase, the high genetic and pathogenic diversity within *V. dahliae* populations and disease-aggravating agronomical practices are some of the factors explaining the difficulties in managing the disease. In this context, an efficient control of VWO can only be achieved under an integrated disease management strategy that combines physical, chemical, biological and agronomical approaches to be implemented both as preventive (pre-planting) and palliative (post-planting) measures [2,3]. Within this strategy, the use of beneficial microorganisms (i.e., biocontrol approaches) represents an interesting, sustainable and environmentally friendly option to be used in combination with other disease control tools.

Different microorganisms have been identified as effective biocontrol agents (BCA) against *V. dahliae* ([2,4,5] and references therein). Thus, just to name the most recent examples, several *Pseudomonas* spp. and *Bacillales* spp. members [6,7], non-pathogenic *Fusarium* strains [8], entomopathogenic fungi such as *Metarhizium brunneum* and *Beauveria bassiana* [9] or arbuscular mycorrhizal fungi [10] have been shown to reduce both the severity of VWO in susceptible olive cultivars and the inoculum density of *V. dahliae* in the soil. Interestingly, several studies have evidenced the importance of the olive-associated microbiota as a reservoir of potential BCAs against VWO [6,7,11,12]. Moreover, a higher abundance of beneficial microbial genera was recently found in the root endosphere community of the resistant cultivar Frantoio, compared to that found in the susceptible cultivar Picual. This also suggests that VWO tolerance can be explained, at least to some extent, by the ability of tolerant cultivars to preferentially recruit beneficial microorganisms and the way constituents of the root endophytic community interact among them [13].

*Pseudomonas simiae* PICF7 (formerly *P. fluorescens* PICF7) [14,15], a natural inhabitant of the olive rhizosphere also able to endophytically colonize the root tissues [16], is perhaps the best characterized BCA against VWO [17,18,19]. However, the underlying mechanisms explaining its endophytic behavior and biocontrol effectiveness remain to be fully understood. For instance, while *P. simiae* PICF7 has been demonstrated to induce systemic resistance against *Botrytis cinerea* in the model plant *A. thaliana* [20], effective control of the olive phytopathogenic bacterium *Pseudomonas savastanoi* pv. *savastanoi* (Psv) was not observed when PICF7 and the pathogen were inoculated at different sites (i.e., PICF7 in the roots and Psv in the stem) [21]. Moreover, although *P. simiae* PICF7 is able to trigger defensive responses in both roots [22] and aerial tissues [17], effective control requires the presence of both the BCA and the pathogen on the same side of an olive split-root study system [18]. Overall, these results suggest that biocontrol performance of PICF7 must rely on a combination of mechanisms like competition for colonization sites, antibiosis and the triggering of local (i.e., in roots) host defense responses.

Efforts to identify PICF7 traits involved not only in VWO biocontrol but also in olive root colonization, including endophytism, were further pursued by Maldonado-González and co-workers [19]. A *P. simiae* PICF7 random transposon-insertion mutant bank was generated and massive screening of transposants was conducted to evaluate whether mutants altered in swimming motility and siderophore (pyoverdine) production had lost biocontrol effectiveness and endophytic colonization ability. Results indicated that these two PICF7 phenotypes were required neither for VWO biocontrol activity nor for the ability to colonize the olive root interior [19]. In order to investigate additional traits potentially involved in root (endophytic) colonization and biocontrol performance of strain PICF7, we focused our attention on selected phenotypes traditionally associated with (i) rhizosphere/soil persistence (i.e., copper resistance), root colonization (i.e., biofilm formation) and plant growth promotion (i.e., phytase activity).

Copper, an essential metal for most organisms, is required in trace amounts as a component of proteins and for cellular processes. However, excessive concentrations of this element can be lethal [23,24]. This makes copper an extremely effective antimicrobial agent, which explains its traditional and extensive use in agriculture. Particularly, most of the fungicides/bactericides used to control some olive diseases are based on copper [25]. Nevertheless, some soil inhabitants have developed/acquired resistance mechanisms towards a range of heavy metals found in soils, including copper, to maintain their fitness and survival capabilities [26]. Beneficial microorganisms able to tolerate significant concentrations of copper could therefore pose advantages from the perspective of controlling olive diseases, including VWO. In silico analysis of the *P. simiae* PICF7 genome previously revealed the presence of at least 11 genes related with copper resistance [14]. Up to date, however, no information is available on the actual tolerance of strain PICF7 to copper. Moreover, whether copper tolerance may confer any advantage with regard to root colonization and VWO biocontrol abilities of PICF7 is unknown.

*Pseudomonas simiae* PICF7 has been earlier demonstrated to promote the growth and enhance the yield of barley (*Hordeum vulgare* L.) plants [27]. Several traits traditionally associated with plant growth promotion have been demonstrated in strain PICF7 (e.g., siderophore production, phytase and phosphatase activities, etc.) [6]. It is plausible to think that a correct nutritional status of the plant may help to better deal with different biotic stresses. Thus, a BCA capable to promote the plant growth by direct mechanisms may also help the host to reach an optimal fitness status, thereby contributing to confront the negative effects caused by the presence of a pathogen in a more efficient way [28]. Phosphorus (P) is an important macronutrient essential for plant growth and development. In soils, 20–80% of phosphate is present in organic forms but the ability of plants to mobilize P from these chemical species is very limited [29]. Some plant growth promoting rhizobacteria (PGPR) are able to convert insoluble phosphates into soluble forms available to plants by the production of phytases [30]. Phytases (phosphatases catalyzing the hydrolysis of phytic acid) release a plant-usable form of P from soils with high content of organic P, a mechanism proposed to promote plant growth [31]. Phytase activity has been reported for PICF7 [6]. Furthermore, in silico analysis of the PICF7 genome has revealed the presence of a gene putatively coding for a 3-phytase [14]. However, no information is available on the possible link between this phenotype and root colonization ability and biocontrol performance of this BCA.

The capability of soil-borne rhizobacteria to exert their beneficial effects on plants firstly depend on their aptitude for colonizing (and persisting on/in) the rhizosphere, the root surface and/or the root interior [28]. Different mechanisms (i.e., production of mucilage, chemotaxis driven by root exudates, bacterial flagella, motility, quorum sensing responses, release of specific biomolecules, cell surface proteins, and site-specific recombinases among others), that can be used either alone or in combination, may be deployed by any given bacterial BCA to ensure successful colonization of the target niche [32,33,34]. Among these mechanisms, several studies have demonstrated the role that biofilm production plays in rhizosphere/root colonization processes [35]. Indeed, in roots colonized by beneficial rhizobacteria, biofilms provide a more protected niche. In addition, they are crucial for living on the roots as well as in the distribution of bacterial cells in this organ [36,37]. Production of bacterial biofilms is a complex process influenced by many genes and factors (e.g., [38,39,40,41]). Biofilms undoubtedly provide advantages to the bacterium able to generate them [42]. So far, nothing is known about the ability of strain PICF7 to produce biofilm. The identification of PICF7 genes involved in this trait, as well as their potential relationships with olive root colonization and biocontrol performance, can thus be useful in explaining the beneficial effects of this endophytic rhizobacteria.

Therefore, and taking into account the previous background, the main objectives of this study were: (i) to identify genes involved in three phenotypes traditionally associated with rhizosphere/soil persistence (copper tolerance), plant growth promotion (phytase activity) and root colonization ability (biofilm formation); and (ii) to determine whether mutants defective in these selected phenotypes could be compromised in the ability of strain PICF7 to effectively colonize olive roots, including the interior of this organ, and to control VWO. Additionally, using full-genome comparison, we aimed to confirm PICF7 as belonging to the *P. simiae* species.

## 2. Results

### 2.1. Comparative Genomics Supports the Taxonomic Assignment of Pseudomonas simiae PICF7

**Figure 1:**
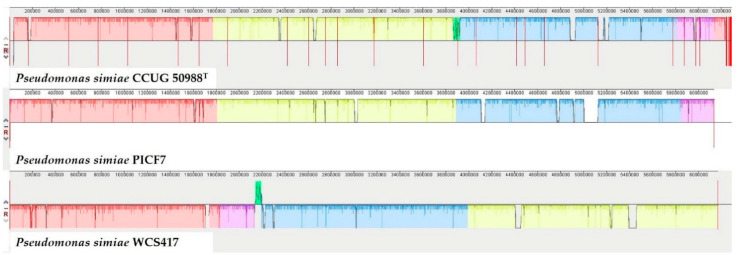
Similarity plot of type strain *Pseudomonas simiae* CCUG 50988^T^, *Pseudomonas simiae* WCS417 and *Pseudomonas simiae* PICF7 constructed by using progressive Mauve (see main text, Section 2.1, for color codes).

A previous multi-locus sequence analysis (MLSA) using concatenated partial sequences of six housekeeping genes showed that strain PICF7 clustered with *Pseudomonas simiae* WCS417 [6]. In order to confirm the taxonomy of PICF7, its full genome was compared with the genomes of *P. simiae* WCS417 [43] and the reference strain *Pseudomonas simiae* CCUG 50988^T^ [44] (Figure 1). Alignment using the MAUVE tool showed that *P. simiae* CCUG 50988^T^, *P. simiae* WCS417 and *P. fluorescens* PICF7 are nearly identical except for some small genomic islands that are unique for each of the strains. The average nucleotide identity (ANI) algorithm using BLAST (ANIb) analysis showed that PICF7 shared 99.38% average nucleotide identity with *P. simiae* CCUG 50988^T^ and 99.35% with *P. simiae* WCS417 (http://jspecies.ribohost.com/jspeciesws/#genomesdb-detail;id=23494 (accessed on 17 January 2020)). The same colors shown in Figure 1 correspond to similar genomic regions present in the three strains. White gaps indicate genomic regions not shared among the analyzed strains. The small genomic region marked in a green color is present in the genomes of *P. simiae* WCS417 and in the *P. simiae* type strain but is missing in PICF7. Thus, results showed a high identity level among the three genomes, confirming strain PICF7 as *P. simiae*.

### 2.2. Screening of a PICF7 Mutant Bank to Identify Altered Target Phenotypes

More than 5.500 Tn5-Tc^R^ (Tetracycline-resistance) insertion mutants (transposants) of a pre-existing random transposon insertion mutant bank [19] were checked for phenotypes altered either in (i) growth on media supplemented with copper, or (ii) biofilm formation, or (iii) phytase activity. The screening of *P. simiae* PICF7 Tc^R^-resistant individual colonies allowed us to generate an initial collection of 63 mutants affected in one of the phenotypes mentioned above.

**Figure 2:**
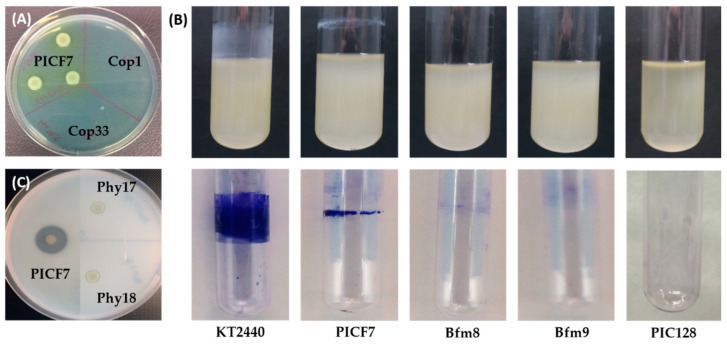
Phenotypes altered in selected *Pseudomonas simiae* PICF7 mutants. (**A**) Cop1 and Cop33 mutants are unable to grow on LB agar plates supplemented with 4.5 mM CuSO_4__·_5H_2_O (image taken 3 days after bacterial inoculation. (**B**) Mutants Bfm8 and Bfm9 are defective in producing biofilm. Upper image shows borosilicate tubes containing the bacterial cultures in LB medium. Lower image shows the result obtained after performing the staining procedure with crystal violet and the inner surface-adhered bacteria. (**C**) Mutants Phy17 and Phy18 are impaired in phytase activity when assayed in Phytase Specific Medium (PSM) (picture taken 3 days after bacterial inoculation). See the Materials and Methods section for full experimental details. PICF7, *P. simiae* PICF7; KT2440, *Pseudomonas putida* KT2440. These strains were used as positive controls. PIC128, *Pseudomonas indica* PIC128 was used as a negative control.

Concerning the identification of mutants defective in copper tolerance, toxicity tests (see de Material and Methods section) using four different media supplemented with CuSO_4_·5H_2_O determined that the tolerance threshold of *P. simiae* PICF7 to copper depended on the medium used. Luria Bertani (LB), Nutrient Agar (NA), Potato Dextrose Agar (PDA) and Standard Succinate Medium (SSM) were used. Regarding NA medium, PICF7 was able to grow up to a copper concentration of 2 mM. However, the tolerance threshold was lower in PDA (0.5 mM) and SSM (0.08 mM) media. Eventually, LB agar and a concentration of 4.5 mM CuSO_4_·5H_2_O were the culturing conditions chosen to identify PICF7 mutants defective in copper tolerance. Thirty-six Tc^R^ transposants presenting alterations either in growth or colony morphology were identified. Only two of them were not able to grow at all (mutants Cop1 and Cop33; Figure 2A). Another three showed an altered colony color, and the rest displayed differences in colony size/morphology compared to the wild-type strain, although all of them were able to grow at the conditions tested. Mutants of the two latter groups were not further investigated in this study.

Regarding to the search for transposants showing a phenotype altered in biofilm formation, and after conducting the screening strategy designed to identify them (see the Materials and Methods section), only five mutants could be identified. Loss in the ability to form a biofilm in liquid medium of the two mutants finally selected (Bfm8 and Bfm9) is shown in Figure 2B. It is worth mentioning that the ability of wild-type PICF7 to produce a biofilm was considerably lower than that of the reference strain (*Pseudomonas putida* KT2440) used to assess this phenotype, at least under our experimental conditions.

With regard to the identification of Tn5-Tc^R^ insertion mutants impaired in phytase activity, a total of 22 mutants that showed no halo formation or reduced haloes around the colonies in Phytase Specific Medium (PSM) were detected. Only two transposants (mutants Phy17 and Phy18; Figure 2C) unable to form haloes, indicating total loss of phytase activity, were eventually selected for further experiments.

Finally, BOX-PCR fingerprinting revealed no modification of the banding pattern in any of the selected *P. simiae* PICF7 mutants, compared to that of the wild-type (data not shown). This result confirmed that all mutants were PICF7 derivatives (i.e., discarding accidental contamination with other bacteria during processing), and that no major alterations were introduced in their genomes upon insertion of the transposon.

### 2.3. Identification of the Disrupted Gene in the Genomes of Selected Mutants of Pseudomonas simiae PICF7

Identification of the disrupted genes as a consequence of the Tn5-Tc^R^ insertion was attempted for 26 out of the 63 preselected mutants. Eventually, only six of them, two per phenotype here under study (i.e., Cop1, Cop33, Bfm8, Bfm9, Phy17 and Phy18, Figure 2) were selected for subsequent olive root colonization and VWO biocontrol assays (see below). This final selection was based on the following criteria: (i) unambiguous and full altered phenotype, (ii) neither alteration in colony morphology nor in growth rates in different culturing media, and (iii) the unequivocal identification of the transposon insertion site. While this was possible for mutants Cop1, Cop33, Bfm8 and Bfm9, the insertion site of mutants impaired in phytase activity (i.e., Phy17 and Phy18) could not be unambiguously identified after repeated attempts. Indeed, multiple, faint and overlapping PCR products (>8) were always obtained for these mutants, even though PCR conditions were modified for several parameters. Finally, selected mutants were checked for the absence of other altered phenotypes for which they were selected.

**Figure 3:**
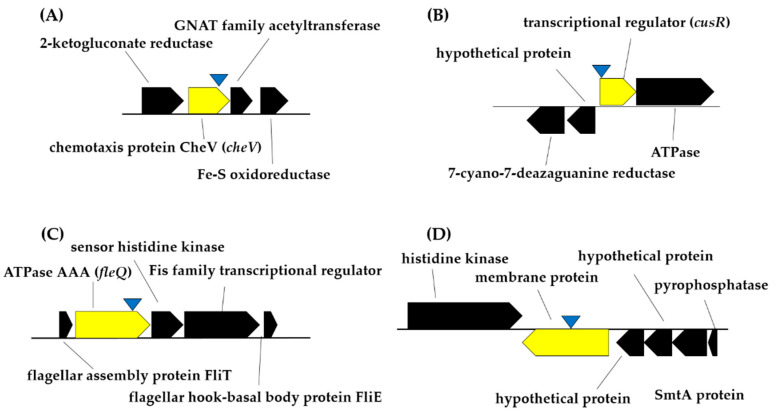
Schemes representing the Tn5-Tc^R^ insertion sites (within genes shown in yellow color) in the genome of the *Pseudomonas simiae* PICF7 selected mutants. (**A**), Mutant Cop1; (**B**), Mutant Cop33; (**C**), Mutant Bfm8; (**D**), Mutant Bfm9. The approximate transposon insertion sites are shown as inverted blue triangles.

Results showed that mutant Cop1 carried the Tn5-Tc^R^ insertion in a *cheV* gene homologue, which codes for a chemotaxis protein, while in mutant Cop33 the insertion was located within the *cusR* gene, potentially coding for a transcriptional regulatory protein that is part of the two-component regulatory system CusS/CusR involved in response to copper and silver (Table 1 and Figure 3). The transposon insertion of mutant Bfm8 was localized in a *fleQ* gene homologue, coding for a flagellar regulatory protein, while in mutant Bfm9 the transposon hit a gene potentially coding for an unidentified 648 aa-long membrane protein (Table 1 and Figure 3). Interestingly, the N-terminal domain of this protein contains putative Flp pilus-assembly TadE/G-like (positions 11–57) and Tad-like Flp pilus-assembly (positions 55–156) motifs.

### 2.4. Only PICF7 Mutants Impaired in Biofilm Formation Are Unable to Internally Colonize Olive Roots

**Figure 4:**
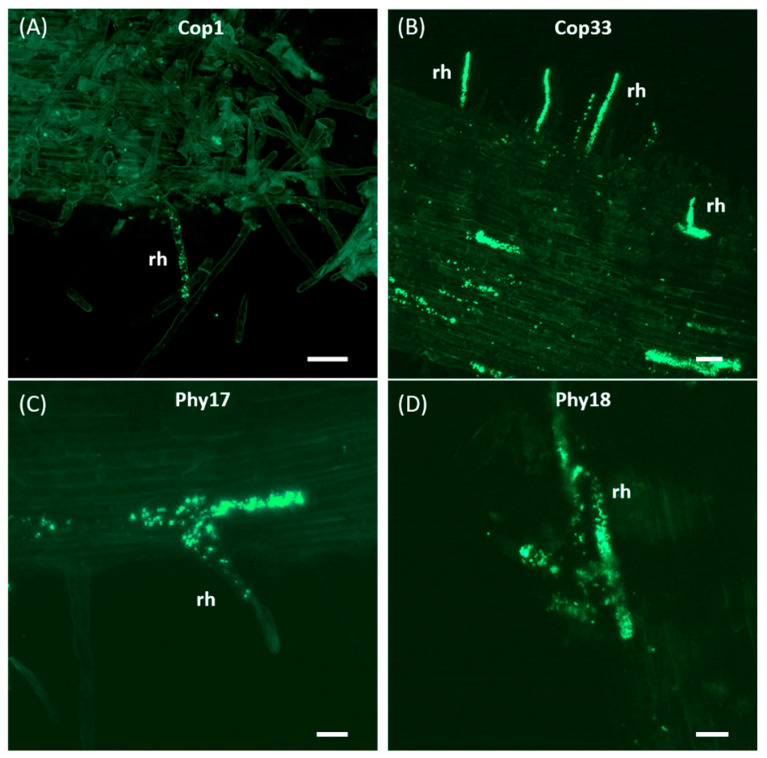
Confocal laser scanning microscopy images of root hairs from olive (cv. Picual) internally colonized by GFP-labeled PICF7 mutants. Images are representative of colonization events photographed from 5 to 10 days after root bacterization. Scale bar represents 50 μm in panels (**A**,**B**), and 20 μm in panels (**C**,**D**). rh, root hair.

Olive roots inoculated with green fluorescent protein (GFP)-labeled derivatives of *P. simiae* PICF7 and of the selected mutants were sampled on a time-course basis (during 20 days after bacterial inoculation; DAI) for confocal laser scanning microscope (CLSM) analysis. Results showed that *P. simiae* PICF7, as well as all analyzed mutants, were able to extensively colonize the olive root surface. At 3 DAI, however, only bacterial cells of mutants altered in copper tolerance (Cop1 and Cop33) and phytase activity (Phy17 and Phy18) were visualized within root hair cells (Figure 4), a characteristic colonization behavior of the wild-type strain PICF7 [45].

**Figure 5:**
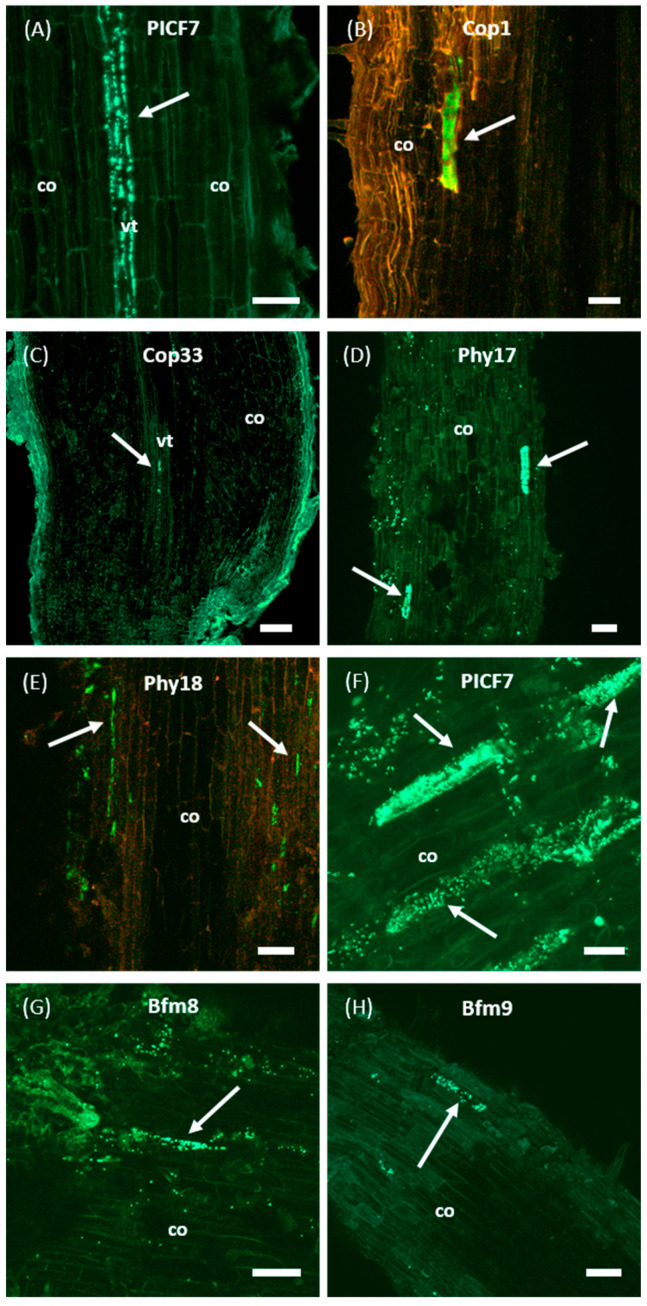
Confocal laser scanning microscopy images of olive (cultivar Picual) roots colonized by GFP-labeled *Pseudomonas simiae* PICF7 and its selected Tn5-Tc^R^ insertion mutants. Images show inner colonization events of different root tissues by wild-type PICF7 (panel (**A**)), and mutants Cop1 (**B**), Cop33 (**C**), Phy17 (**D**) and Phy18 (**E**). Additionally, colonization of olive root surface by wild-type PICF7 (panel (**F**)) and biofilm mutants Bfm8 (**G**) and Bfm9 (**H**) are also shown. Images are representative of the colonization events observed and were taken from 4 to 17 days after root bacterization with fluorescently labelled derivatives. Scale bar represents 50 μm in all panels, except in ((**C**), 100 μm) and ((**F**), 20 μm). White arrows point to spots or microcolonies of the inoculated bacteria. co, cortical cells; vt, vascular tissue. In panels (**B**,**E**), the red channel was added to increase plant tissue contrast and improve visualization.

Longitudinal sections of roots also showed fluorescent bacterial cells colonizing the interior of different root tissues like the intercellular spaces of the root cortex and the vascular system, as well as within epidermal cells (Figure 5A–E). The same colonization pattern was consistently observed for wild-type PICF7 and mutants defective in copper tolerance (Cop1 and Cop33) and phytase activity (Phy17 and Ph18) in a fair number of samples (at least 6 plants were examined for these treatments to obtain conclusive evidence of colonization). Mutants impaired in biofilm formation (Bfm8 and Bfm9), however, were only found on the root surface (Figure 5G,H). Indeed, after two independent experiments no evidence of root endophytic colonization by these mutants could be found in any of the plants examined (12). Thus, according to the results obtained, mutants Bfm8 and Bfm9 have lost the ability to internally colonize the olive root tissues, at least under the experimental conditions used.

### 2.5. PICF7 Mutants Defective in Phytase Activity Show a Significant Reduction of In Vitro Antagonism against Verticillium dahliae

**Figure 6:**
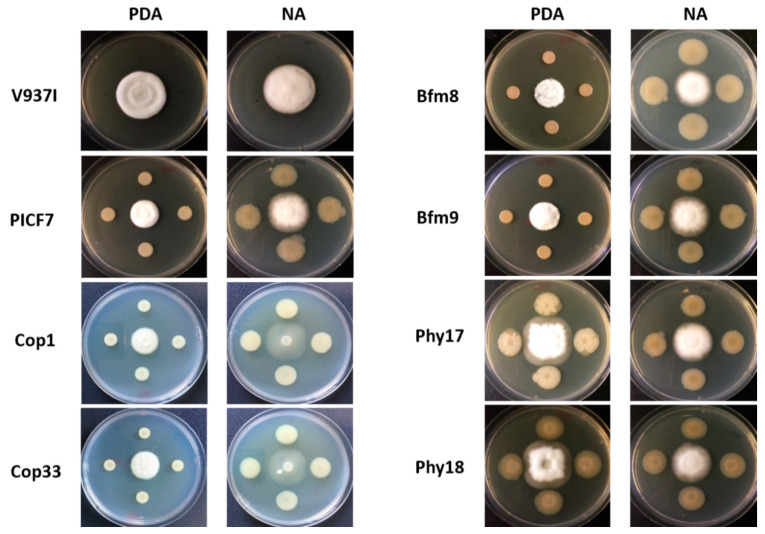
In vitro antagonistic activity of *Pseudomonas simiae* PICF7 and its mutants against *Verticillium dahliae* V937I. Pictures were taken 10 days after incubation at 25 °C. V937I, *Verticillium dahliae* V937I; PICF7, *Pseudomonas simiae* PICF7; PDA, Potato Dextrose Agar; and NA, Nutrient Agar. Images are representative plates used in two independent experiments (see Table 2).

Results from in vitro antagonism assays showed that phytase-deficient mutants Phy17 and Phy18 were not able to inhibit the growth of the defoliating (D) pathotype isolate *V. dahliae* V937I on PDA medium. In experiment I, no inhibition of *V. dahliae* by these mutants was observed (Table 2). In experiment II, only very slight inhibition of the pathogen’s growth (0.51% by Phy17 and 1.03% by Phy18) was scored compared with the wild-type PICF7 (control) in PDA medium (Table 2). In contrast, no significant differences were found between the antagonist behavior of these mutants and that of PICF7 when NA plates were used (Table 2 and Figure 6), indicating the influence exerted by the culturing media (and therefore nutrients availability) over the results scored for this type of experiment.

Regarding biofilm-impaired mutants (Bfm8 and Bfm9), results showed a significantly (*p* < 0.05) higher *V. dahliae* growth inhibition exerted by mutants Bfm8 (Experiment I, both culturing media) and Bfm9 (Experiment II, only in PDA) compared to inhibition percentages scored for the wild-strain PICF7. However, results scored for these mutants varied between experiments. Finally, mutants unable to grow in culturing media amended with copper, Cop1 and Cop33, did not show significant differences in their ability to inhibit *V. dahliae* growth compared to the wild-type strain (Table 2). It is worth mentioning that *V. dahliae* displayed altered mycelium growth on NA plates when Cop mutants were present. This phenomenon was not observed for the other interactions here examined (Figure 6).

### 2.6. PICF7 Mutants Are Not Affected in Biological Control Ability against Verticillium Wilt of Olive

Two independent experiments were carried out to test the biocontrol ability of the six selected *P. simiae* PICF7 mutants. Results showed that mutants Bfm8 and Bfm9 (impaired in biofilm formation), Cop1 and Cop33 (copper sensitive) and Phy17 and Phy18 (lacking phytase activity) displayed similar biocontrol effectiveness against VWO compared to the wild-type strain. Non-inoculated plants (control treatment) and plants inoculated only with the bacterial strains showed normal growth in both bioassays. The first VWO symptoms appeared three weeks after pathogen inoculation in the disease control treatment. Plants initially showed chlorosis and inward rolling of leaves in some cases. Approximately one-month after *V. dahliae* inoculation, the first events of green leaf fall were observed, a symptom that progressed over time until complete defoliation and death of some plants. In fact, mortality reached 25% in Experiment I and 41.67% in Experiment II (Table 3).

In Experiment I, PICF7-bacterized plants displayed significant (*p* < 0.05) reduction of the final mean severity symptoms (FS) and the area under disease progress curve (AUDPC) disease parameters in comparison to non-bacterized (control) plants (Table 3). Besides, a decrease in disease intensity index (DII), disease incidence (DI) and mortality (M) values were also observed (Table 3). Plants inoculated with selected mutants showed a similar behavior to that observed in plants inoculated with the wild-type PICF7 (Table 3). No dead plants were observed for Bfm8, Bfm9, Phy17 and Cop33 treatments.

A more severe VWO epidemic was scored in Experiment II (Table 3), as clearly reflected by M, DII and FS parameters. While in this experiment *P. simiae* PICF7 did not display significant biocontrol activity against VWO, all disease parameters were lower than those scored for the control treatment. Interestingly, however, all mutants here examined were able to effectively control the disease as evidenced by a significant (*p* < 0.05) reduction of the AUDPC compared to non-bacterized plants. FS and DII parameters were also reduced in all mutant treatments (Table 3). Despite mutants Phy17 and Phy18 were altered in their capacities to antagonize *V. dahliae* (Table 2; Figure 4), biocontrol ability was not compromised. In addition, Phy mutants showed the lowest values for FS, FDII and AUDPC (Table 3) parameters in experiment II. Altogether, results from both experiments evidenced that the three phenotypes here under evaluation are not determinants of VWO biocontrol by *P. simiae* PICF7.

## 3. Discussion

The success of BCA relies on different modes of action. Competition for nutrients and space, niche colonization ability, induction of local and/or systemic host defense responses, and antibiosis are some of them, which can work individually or in combination [5,28,46]. *Pseudomonas simiae* PICF7 (formerly *P. fluorescens* PICF7) [14,15] is effective in controlling Verticillium wilt under different experimental conditions, and not only in olive [15,17,19] but also in the model plant *Arabidopsis thaliana* [20]. Yet, the underlying mechanisms involved in VWO control are insufficiently understood. Moreover, they could be diverse and operate differentially in time and space [17,18,19].

Earlier, by the MLSA of concatenated partial sequences of housekeeping genes, we found that strain PICF7 clustered with the model BCA *P. simiae* WCS417 [6]. This latter strain was taxonomically reclassified (originally *P. fluorescens*) by Berendsen and co-workers [43]. Recently, Pieterse and co-workers [47] suggested that other strains formerly classified as *P. fluorescens*, such as PICF7, may actually be part of the *simiae* group based on genome sequence identities. This has been confirmed by the full-genome comparison analysis performed in this work. Indeed, results showed that strain PICF7 showed more than 99.3% ANIb with both *P. simiae* CCUG 50988^T^ and *P. simiae* WCS417. Actually the three strains are nearly isogenic but for some genomic islands, probably as the result of horizontal gene transfer, found to be unique for each studied strain.

A previous study evaluated whether siderophore (pyoverdine) production and swimming motility were involved in the effectiveness of PICF7 to control VWO. By mutant analysis both phenotypes were discarded as needed for such a benefit since defective mutants were able to control the disease to the same extent as the wild-type strain [19]. In order to further understand the molecular bases conferring *P. simiae* PICF7 the ability to control VWO, as well as to colonize olive roots, additional phenotypes have been evaluated in this work by using the same mutant analysis approach.

A limiting factor of plant growth is the usually low P availability in soils. To alleviate this limitation, huge amounts of chemical fertilizers are used every year. Some microorganisms have developed the ability to solubilize mineral phosphates fixed in the soil [48]. Thus, through mechanisms such as the activity of phosphatases and phytases, which release P from organic phosphates, or the exudation of organic acids to solubilize inorganic phosphate, P is made available to plants [49]. Microorganisms with the ability to mobilize phosphate are of great interest for crop improvement, soil management and agro-biotechnological applications. Interestingly, and related to our aims, many of them also have the capacity to suppress diverse phytopathogens [6,50,51,52]. Earlier, in silico analysis of the PICF7 genome allowed the identification of a putative 3-phytase-coding gene [14]. Moreover, phytase activity has been confirmed for wild-type PICF7 ([6]; this study). Only two fully phytase-defective PICF7 mutants, Phy17 and Phy18, were found after the screening of more than 5500 Tn5-Tc^R^ transposants. Unfortunately, the transposon insertion site could not be unequivocally determined in any of them, although insertions seemed to be localized at different positions in the PICF7 genome according to the distinctive PCR profiles obtained for each mutant (data not shown). Whether a truly functional phytase-3 gene is present in PICF7, or whether alternatively other genes are involved in phytase activity, could not thus be unveiled. A site-directed mutagenesis strategy will be needed to achieve this goal. However, phytase activity was fully abolished in the two selected mutants, allowing the assessment of this phenotype as for its involvement in root colonization and VWO biocontrol. Both mutants lost their ability to inhibit the growth of *V. dahliae* (a representative of the D pathotype), although results varied depending on the culturing medium used. Such medium-dependent differences have been previously observed when characterizing a collection of olive-root-associated rhizobacteria [6,7,53]. While competition for nutrients and/or in vitro antibiosis towards *V. dahliae* seemed to be affected in these mutants, results from biocontrol experiments clearly showed that Phy17 and Phy18 controlled VWO to the same extent that the wild-type strain did. In addition, both phytase-defective mutants were able to colonize the interior of olive roots. Thus, results allowed discarding this phenotype as relevant for endophytic lifestyle and for biocontrol of *V. dahliae* by PICF7.

Successful biocontrol of soilborne fungal diseases must be preceded by efficient and persistent colonization of the niche (e.g., rhizosphere, rhizoplane, root interior, etc.) where BCA exert their beneficial effects [34,54]. The ability to form biofilms on plant roots has been reported as an important trait linked to the colonization ability of many biocontrol rhizobacteria. For example, the effectiveness to suppress *Phytophthora capsici* in pepper plants by *Pseudomonas corrugata* CCR04 and CCR80 is linked to good colonization of the host plant that is facilitated by the ability of both strains to form biofilms [55]. Similarly, the mutant 1srfAB, affected in biofilm formation, had a reduced colonization ability of melon roots and was impaired in biocontrol efficacy against bacterial fruit blotch compared with the wild-type *B. subtilis* 9407 [56]. Finally, *Bacillus subtilis* mutants enhanced in biofilm formation improved their biocontrol efficacy against Ralstonia wilt on tomato plants compared to biofilm-defective mutants [54]. Nevertheless, these authors also suggested that biofilm formation alone could not be enough to explain biocontrol activity. This was based on the observation that some wild-type strains of *Bacillus* producing a robust biofilm showed poor biocontrol performance. These results highlight the difficulties in establishing a clear relationship between the biofilm production process and effective biocontrol.

While the link between biofilm formation, root colonization ability and biocontrol performance has been demonstrated in different studies [42,55,57], our results indicate that this relationship cannot be established to explain VWO control by PICF7, particularly as for endophytic colonization is concerned. Indeed, although the two mutants impaired in biofilm formation (Bfm8 and Bfm9) displayed the same olive root surface colonization behavior than that of PICF7, they had lost their ability to colonize the root interior. However, both mutants displayed the same in vitro antagonist capacity against *V. dahliae* and biocontrol performance against VWO as that observed for the wild-type strain. Strain PICF7 was earlier demonstrated to control Verticillium wilt in *A. thaliana* even though no evidence of endophytism was found in the model plant [20]. Thus, results here obtained point to the fact that inner colonization of olive roots by PICF7 cells is not needed for the effective biocontrol of *V. dahliae*. Therefore, most of the biocontrol activity appears to rely on PICF7 activity on the olive root surface (e.g., attachment to pathogen hyphae [58], antibiosis, triggering of host local defense responses [22]), although competition for nutrients and space cannot be excluded either. In summary, biofilm formation by PICF7 is not needed for biocontrol activity but is important for colonization of the root interior.

Production of bacterial biofilms is a highly complex process in which many genes, as well as environmental factors, are involved. In silico analysis of the PICF7 genome revealed the presence of different gene clusters presumably involved in biofilm formation such as *pelABCDEFG, pslADEFGHJKL* and *pgaABD* [14]. The implication of *pel*, *psl* and *pga* genes in biofilm-forming bacteria has been described elsewhere [59,60,61,62]. However, mutants Bfm8 and Bfm9 here reported were disrupted in genes other than the previous ones, unveiling that additional loci in the PICF7 genome are relevant for biofilm formation. Thus, mutant Bfm8 harbored the Tn*5*-Tc^R^ insertion in a *fleQ* gene homologue, coding for a flagellar regulatory protein. This protein has been identified as a master regulator of flagella and biofilm formation in *Pseudomonas fluorescens* F113 and *Pseudomonas putida* KT2440 [63]. Mutations affecting *fleQ* produced non-motile, non-flagellated cells in the phytopathogenic bacterium *Pseudomonas syringae* pv. tomato DC3000 [64] and in the rhizobacterium *P. putida* KT2442 [65]. Disruption of *fleQ* seriously compromised biofilm formation in the latter strain as well as in *P. putida* KT2440 [66]. Finally, the transcriptional regulator FleQ has been described for its role in the modulation of *lapA* and *bcs* genes expression, involved in the production of biofilm matrix components in this latter strain [66]. The Tn*5*-Tc^R^ insertion in mutant Bfm9 was localized in a gene potentially encoding an unidentified membrane protein. One of the first stages in biofilm formation is mediated by the formation of an active attachment of bacterial apexes (fimbriae, flagella or pili) to the host surface. Interestingly, the membrane protein whose coding gene was disrupted in mutant Bfm9 contains putative Flp pilus-assembly TadE/G-like and Tad-like Flp pilus-assembly motifs (SSDB Motif Search Result: pff:PFLUOLIPICF705200 (kegg.jp)). *Tad* (*t*ight *ad*hesion) genes have been described for their role in biofilm formation. In addition, they have been shown to play an important role in colonization and pathogenicity in some bacterial species (i.e., *Aggregatibacter* (“Actinobacillus”) *actinomycetemcomitans*, *Pseudomonas aeruginosa*) [67,68]. Recently, Blanco-Romero and co-workers [69] have extensively studied the phylogenetic distribution of Flp/Tad pili. They reported that the required machinery for their biosynthesis is often found in plant-associated beneficial bacteria, with a likely involvement in rhizosphere colonization processes. Moreover, the *Flp* gene and the *tad* locus are linked, the latter encoding a macromolecular machine needed for the assemblage of type Ivb Flp subfamily adhesive pili [70]. Type IV pili have been described by their implication in biofilm formation [71,72]. Finally, it is worth mentioning that CLSM imagery has proven that PICF7 is able to form biofilms on/in roots of olive ([45,58]; this study) and cereal species [27]. However, biofilms were usually observed at localized spots. It might well be that PICF7 does not have a high capacity to produce biofilm compared to other robust biofilm producers such as *P. putida* KT2440 (Figure 2).

Different genes related to copper resistance, including copper tolerance two-component regulatory system *cusSR* (5) and copper resistance protein-encoding genes *copBCD* and *copDC*, were earlier identified in silico in the *P. simiae* PICF7 genome [14]. In our study, a gene potentially coding for a chemotaxis protein (CheV) has also been identified to be involved in copper tolerance, as revealed by the phenotype altered in mutant Cop1. Chemotaxis enables, for instance, bacteria to move towards nutrient sources (positive chemotaxis) or away from toxins (negative chemotaxis) [73]. In this way, chemotaxis is closely related to numerous biological processes that imply the movement of bacterial cells such as biofilm, cell aggregation or plant colonization ([74] and references therein). The actual link between CheV and copper tolerance by PICF7 needs further analysis beyond the aims of this study. Regarding Cop33, the transposon insertion was localized in a *CusR* gene that encodes a copper-responsive two-component system (TCS). Bacterial TCS allow perceiving, responding and adapting to environmental stimuli. It has been reported that the Cus system is involved in the homeostasis of copper, which is activated in the presence of submillimolar concentrations of this metal [75]. In *Escherichia coli*, the sensor kinase CusS forms a TCS with the response regulator CusR. This TCS has been demonstrated to be required for copper and silver resistance in this bacterium [76]. Furthermore, the CusR/CusS system is essential for the regulation of the *cusCFBA* operon, responsible for exporting both metals outside the cells [76,77].

Copper appears as one of the most common contaminants in soils [78]. In agriculture, most of the antifungal chemicals traditionally used against many phytopathogens are based on copper. Contamination and exposure to high doses of heavy metals is therefore a serious problem affecting agricultural fields [79]. The combination of BCA able to tolerate copper with reduced amounts of copper-based biocides may lessen the inputs of the latter to the environment, resulting in an interesting and more sustainable practice to control phytopathogens. Among *Pseudomonas* spp., including both beneficial and phytopathogenic, copper tolerance level may be highly variable [26,80]. In our study, *P. simiae* PICF7 was found to tolerate copper concentrations up to 4.5 mM of CuSO_4_·5H_2_O in LB, in agreement with values described elsewhere for a copper-resistant *Pseudomonas extremaustralis* [81]. Similarly, the PGPR *Pseudomonas psychrotolerans* CS51 also tolerates concentrations of 1mM of copper. Interestingly, cucumber plants pre-inoculated with strain CS51 showed an enhanced tolerance to copper compared with non-bacterized plants [82]. This offers interesting perspectives in the use of beneficial metal-tolerant rhizobacteria to enhance plant tolerance to heavy metals, coupled with additional plant growth-promoting traits present in these microorganisms [83].

## 4. Materials and Methods

### 4.1. Culturing Media and Conditions, Microorganisms and Plasmid

Bacterial strains, mutant derivatives, the fungal pathogen and the plasmid used in this study, including main characteristics and sources/references, are listed in Table 4.

Starting cultures of *Bacillus* sp. PIC28 [7], *Pseudomonas indica* PIC128, *P. putida* KT2440, and the wild-type *P. simiae* PICF7 were grown overnight in LB medium (5 g yeast extract, 10 g triptone and 5 g NaCl in 1000 mL of distilled water) at 28 °C. GFP-labelled derivatives of the wild-type *P. simiae* PICF7 (*P. simiae* PICF7 (pLRM1)) and of the six selected mutants (Cop1 (pLRM1), Cop33 (pLRM1), Bfm8 (pLRM1), Bfm9 (pLRM1), Phy17 (pLRM1) and Phy18 (pLRM1)) (Table 4), were used in order to evaluate olive root colonization ability by confocal CLSM. In this case, bacteria were grown at 28 °C in LB amended with gentamicin (Gm) and/or tetracycline (Tc) at a concentration of 50 mg/L or 20 mg/L, respectively. *Escherichia coli* DH5α harboring plasmid pLRM1 [85] (Table 1) was grown on LB amended with Gm (50 mg/L) at 37 °C.

Bacterial inocula were always adjusted at 1·10^8^ cfu/mL by spectrophotometer measurement (A600 nm) and by building up standard curves. Besides, for root colonization assessment, in vitro antagonism assays and greenhouse biocontrol experiments, the actual number of viable cells of each working inoculum was checked by plating serial dilutions on LB agar plates (PICF7 wild-type colonies), LB agar plates plus Tc (PICF7 Tn5-Tc^R^ mutant derivatives), LB agar plates supplemented with Gm (PICF7 GFP-labeled derivative) or LB agar plates supplemented with Tc and Gm (GFP-labeled PICF7 mutant derivatives) (Table 4).

The fungal pathogen *V. dahliae* V937I was grown on PDA (Oxoid, Basingstoke, UK) plates at 25 °C in the dark. Subsequently, the required inoculum was prepared as indicated in the appropriate sections below. All microorganisms are deposited in the culture collection of the Laboratory of Plant-Microorganism Interactions, Crop Protection Department, Institute for Sustainable Agriculture (IAS, Córdoba, Spain).

### 4.2. Comparative Analysis of Pseudomonas simiae Genomes

Strains PICF7 and WCS417 were previously reported as phylogenetically related by comparing concatenated partial sequences of six housekeeping genes [6]. In order to confirm whether strain PICF7 (formerly classified as *P. fluorescens*) belonged to the *P. simiae* species, full genome comparisons were performed with progressive MAUVE [87]. Thus, the genomes of two representatives *P. simiae* strains, namely CCUG 50988^T^ type strain [44] and *P. simiae* WCS417 [43], were selected and compared with the genome of strain PICF7 [14]. Prior to alignment, contigs of *P. simiae* WCS417 and *P. simiae* CCUG50988^T^ genomes were reordered with PICF7 strain as the reference genome using the designated MAUVE tool.

### 4.3. Screening of Pseudomonas simiae PICF7 Mutants

A pre-existing random transposon (Tn5-Tc^R^) insertion mutant bank of *P. simiae* PICF7 [19] was used in this study. Altered phenotypes in copper resistance (potentially involved in soil/rhizosphere persistence), biofilm formation (putatively related to colonization ability) and phytase activity (potentially linked to plant growth promotion) were ad hoc evaluated. The screening of more than 5.500 Tc^R^ colonies allowed the identification of different mutants (transposants) for each of the phenotypes under evaluation. Additionally, selected mutants were further tested to confirm that they were affected neither in growth nor in any of the other phenotype under study.

#### 4.3.1. Identification and Selection of Mutants Impaired in Copper Tolerance

Overnight cultures (28 °C, 175 rpm) of *P. simiae* PICF7 Tc^R^ transposants grown in 96-well microplates containing liquid LB were spotted using sterile toothpicks on LB square plates (12 × 12 cm) amended with CuSO_4_·5H_2_O 4.5 mM (see below), along with the parental strain PICF7 that was used as a control. Previously, a toxicity test was conducted to determine the tolerance threshold of wild-type PICF7 to CuSO_4·_5H_2_O. A stock solution (0.5 M) was prepared by dissolving CuSO_4_·5H_2_O in distilled water. This stock was then sterilized by filtration using a 0.2-µm pore size nylon membrane. One fresh individual colony of *P. simiae* PICF7 grown on LB agar was resuspended in 10 mM MgSO_4_·7H_2_O. Subsequently, bacterial cells (100 µL) were plated on LB, NA (Oxoid), PDA and SSM (per liter: 6 g K2HPO_4_, 3 g KH_2_PO_4_, 1 g (NH_4_)_2_SO_4_, 0.2 g MgSO_4_·7H2O, 4 g succinic acid, 15 g agar; pH 7) containing CuSO_4_·5H_2_O at increasing concentrations, ranging from 0.08 to 20 mM. Plates were then incubated at 28 °C and bacterial growth was checked for at least 4 days. This assay was performed twice with three replicates (plates) per culturing media and CuSO_4·_5H_2_O concentration. Eventually, LB medium amended with CuSO_4·_5H_2_O 4.5 mM was used for mutant selection.

#### 4.3.2. Identification and Selection of Mutants Impaired in Biofilm Formation

In order to test the capacity of biofilm formation, PICF7 transposants were grown in LB liquid medium (28 °C, 175 rpm) in 96-well microplates for a first bulk screening. Biofilm production was analyzed following the indications, slightly modified, by Huertas-Rosales and co-workers [88]. After 8 h of incubation, bacterial cultures were removed and non-adhered cells were washed away by rinsing the wells with distilled water. Biofilm was stained with 0.1% crystal violet for 20 min. After that, the dye was removed and distilled water was added into each well after 5 min. Then, wells were rinsed twice with distilled water and dried at room temperature. After this initial massive screening, selected mutants unable to form a biofilm were tested individually in borosilicate glass tubes at least three times with three technical replicates. For this, each mutant was grown overnight in liquid LB medium (28 °C, 175 rpm). Subsequently, aliquots were transferred to new borosilicate glass tubes and cell suspensions were adjusted to 0.05 (A600 nm). Tubes were then incubated at 28 °C, 40 rpm. After 8 h, bacterial cultures were removed and the staining protocol described above was applied. *Pseudomonas putida* KT2440 [84], kindly provided by Dr. María José Soto Misffut (Estación Experimental del Zaidín, CSIC) and *P. simiae* PICF7 wild-type strain, were used as positive controls, while strain *P. indica* PIC128 from Laboratory of Plant-Microorganism Interactions (IAS-CSIC) was used as a negative control in all assays (Table 1).

#### 4.3.3. Identification and Selection of Mutants Defective in Phytase Activity

*Pseudomonas simiae* PICF7 transposants (>5.500) were grown overnight in LB liquid medium amended with Tc in 96-well microplates at 28 °C, 175 rpm. Subsequently, each culture was spotted individually using sterile toothpicks on square plates (12 × 12 cm) containing a modified PSM [89] (per liter: 10 g glucose, 2 g CaCl_2_, 5 g NH_4_NO_3_, 0.5 g KCl, 0.5 g MgSO_4_·7H_2_O, 0.01 g FeSO_4_7H_2_O, 0.01 g MnSO_4_·7H_2_O, 4 g Na Phytate, 15 g agar; pH 7). Wild-type PICF7 colonies were used as a positive control since phytase activity was previously confirmed for our model strain. The ability to hydrolyze sodium phytate (positive result) is visualized by the production of clear halo around the bacterial colony. *Bacillus* sp. PIC28 was used as a negative control. Mutants defective in phytase activity identified in this first bulk screening were individually re-checked later on by placing a 10 μL-droplet of a bacterial culture grown overnight on the center of a plate (9 cm of diameter) with PSM medium amended with Tc. Plates were incubated at 28 °C and checked during at least 3 days for the presence of a clear halo. This experiment was performed twice with three technical replicates.

### 4.4. Identification of Transposon Insertion Sites in Selected PICF7 Mutants

Total DNA from 63 preselected mutants showing alteration in one of the three phenotypes here under study was extracted and purified using the JetFlex Genomic DNA Purification Kit (Corporate Headquarters Genomed Gmbh, Löhne, Germany). After that, and in order to discard potential contamination, BOX-PCR fingerprinting was conducted to verify the presence of the same amplification pattern in mutants that were obtained for the wild-type strain. Amplifications were carried out using the BOX A1R (5′-CTACGGCAAGGCGACGCTGACG-3′) primer [90,91]. PCR conditions were as follows: initial denaturation step at 95 °C for 6 min; 35 cycles of denaturation at 94 °C for 1 min; annealing at either 53 °C for 1 min; extension at 65 °C for 8 min; and a final extension at 65 °C for 16 min. PCR products were analyzed in 1.5% agarose gels stained with RedSafe and visualized with a VersaDoc Imaging System (Bio-Rad Laboratories, Inc., USA). Two clones of each selected transposant were tested and cryopreserved in 30% glycerol at −80 °C.

The identification of the disrupted gene due to Tn5 insertion was performed on a selection of mutants showing neat alteration of the target phenotype. Thus, 26 out of the 63 preselected mutants were eventually analyzed by a combination of arbitrary and nested-PCR strategy previously described by Maldonado-González and co-workers [19]. When needed, PCR parameters were empirically adjusted to obtain neat, single PCR products. Amplification products were electrophoresed in 0.8% agarose gels and visualized under UV light. The observed amplicons were extracted from gels and purified using Megaquick-spin Total Fragment DNA purification Kit (iNtRON Biotechnology, Sangdaewon-Dong, South Korea). The region adjacent to the insertion site of Tn5 for each mutant was sequenced (Sistemas Genómicos S.L., Valencia, Spain) using primer Tn5Int. DNA sequences were then compared against the complete PICF7 genome sequence (GenBank, INSDC CP005975) and nucleotide databases using BLASTn and BLASTx algorithms in the NCBI website (https://www.ncbi.nlm.nih.gov/ (accessed on June 2020)).

### 4.5. Evaluation of the Olive Root Colonization Ability by Pseudomonas simiae PICF7 Mutants

In order to assess whether selected PICF7 derivative mutants (Table 4) displayed the same root colonization pattern as that of the wild-type strain, fluorescently labelled derivatives were constructed as previously described by Maldonado-González and co-workers [19]. The selected Tn5-Tc^R^ mutants were transformed with plasmid pLRM1 (Gm^R^, GFP) (Table 4) [85]. For this, electrocompetent cells of each mutant were prepared based on protocol described by Miller and Nickoloff, 1995 [92], keeping Tc selective pressure in the cultures. The “Plasmid Extraction Mini” kit (Favorgen Biotech Co., Ping Tung, Taiwan) was used to purify plasmid pLRM1 from *Escherichia coli* DH5α. Ice-thawed aliquots (40 µL) of electrocompetent cells previously prepared was mixed with 2 µL of purified plasmid DNA in prechilled 1.5 mL microfuge tubes. Plasmid pLRM1 DNA was electroporated into electrocompetent cells of each selected mutant as described by Prieto and Mercado-Blanco [16]. Then, cell suspensions were plated onto LB plates amended with Tc and Gm and incubated at 28 °C. Three individual Tc^R^/Gm^R^ colonies of each mutant were randomly chosen. The presence of plasmid pLMR1 in the selected transformants was checked by plasmid purification and restriction analysis with *Sac*I, *Xba*I and *Bam*HI (Thermo Fisher Scientific, Waltham, MA, USA). Additionally, bacterial fluorescence was confirmed by visualizing bacterial cells under a Nikon Eclipse 80i epifluorescence microscope (Nikon Instruments Europe BV, Amstelveen, The Netherlands). Selected transformants were cryopreserved in 30% glycerol at −80 °C.

The root colonization ability of GFP-labeled derivatives of the selected mutants was then evaluated. A fluorescently labelled derivative of strain PICF7 harboring the plasmid pLRM1 [19,85] (Table 4) was used as control. Selected PICF7 mutants were grown at 28 °C and 180 rpm in LB liquid medium supplemented with Gm and Tc. Bacterial cells of GFP-labeled mutants and PICF7 wild-type strains were centrifuged at room temperature (11,000 *g*, 1 min) and the spent medium was discarded. The cell pellets were resuspended in 10 mM MgSO_4_·7H_2_O and bacterial concentration in the suspension was determined by measuring absorbance at 600 nm using standard curves for each mutant. Prior to the inoculation of olive plants, the fluorescence of bacterial cells was confirmed by a Nikon Eclipse 80i epifluorescence microscope.

A total of 48 olive plants (3-month-old, cv. Picual) purchased at a commercial nursery located in Córdoba province (Southern Spain) were used in this experiment. The bioassay consisted of 8 treatments (6 plants per treatment) corresponding to each of the 6 mutants finally characterized and selected (Table 1), *P. simiae* PICF7 wild-type (positive control) and a manipulation control (non-inoculated plants). Olive plants were carefully uprooted from the original substrate and roots were gently washed under tap water. The entire root systems were then inoculated by dipping them into a 300 mL of a bacterial suspension (1·10^8^ cfu/mL in 10 mM MgSO_4_·7H_2_O) of each strain for 30 min. Roots of the non-inoculated plant treatment were just immersed in 300 mL of 10 mM MgSO_4_·7H_2_O. After that, plants were transferred into 9 × 9 × 10 cm polypropylene pots containing the same potting substrate used in the nursery. Finally, each plant was watered with 45 mL of the same inoculum immediately after planting. Plants were then maintained in a growth chamber at controlled conditions (23 ± 1 °C, 60–90% relative humidity). In order to reduce the stress of the plants after manipulation, inoculation and transplanting processes, the photoperiod was gradually increased over 5 days until it reached 14 h daylight.

Roots inoculated with GFP-labeled mutants and wild-type strain were examined at 3, 4, 5, 10, 17 and 20 DAI. Soil particles adhered to the root system were removed by shaking the roots. After that, root segments (1–4 cm long) representative of the entire root system of each sample were collected. Roots were washed with distilled water and visualized under Axioskop 2MOTmicroscope (Carl Zeiss Inc, Jena GmbH, Oberkochen, Germany) set with a krypton and an argon laser, controlled by a Carl Zeiss Laser Scanning System LSM5 PASCAL software (Carl Zeiss Inc). The software Zeiss LSM Image Browser version 4.0 (Carl Zeiss Inc) was used for imaging and post-processing of the confocal stacks and maximum projections.

In order to better understand localization of GFP-labeled bacterial cells, longitudinal sections of root segments (about 30 micrometers thick) were obtained using a Vibratome Series 1000plus (TAAB Laboratories Equipment, Aldermarston, UK) and analyzed similarly. A fair number of root samples were examined until we obtained clear evidence of both external and internal root colonization. In the case of mutants Bfm8 and Bfm9 the experiment was performed twice in order to confirm results obtained in a first bioassay.

### 4.6. In Vitro Antagonism Assays

Antagonism of *P. simiae* PICF7 mutants against the olive D isolate *V. dahliae* V937I (Table 4) was evaluated using dual-culture in vitro assays in PDA and NA media. Isolate V937I was grown during seven days on PDA plates at 25 °C. Spore suspension was prepared from seven-day-old culture by adding 5 mL of sterile distilled water to a petri dish and scraping off the cultures with a Digralsky spreader. The inoculum concentration was adjusted to 1·10^6^ conidia/mL with a Neubauer hemocytometer. *V. dahliae* V937I conidia (10-µL) were placed in the center of the plates. Cultures of *P. simiae* PICF7 and their mutants were prepared and adjusted as mentioned above (see Section 4.1). Subsequently, each plate was inoculated with four equidistant drops (10 µL per drop) of a bacterial suspension placed at 2.5 cm from the center of the plate. Plates containing only an individual drop of conidia suspension were used as control reference of *V. dahliae* growth in the absence of bacteria. The growth of the fungal colonies was measured after 10 days of incubation at 25 °C in the dark. The inhibition of the pathogen growth (relative inhibition index, PI) was expressed as a percentage according to the equation:

$$\mathrm{PI}\;=\;\left(\frac{Rc-Ra}{Rc}\right)\;\times \;100$$

where *Rc* is the average radius of the *V. dahliae* colony in the absence of antagonist bacterium (cm) and *Ra* is the average radius of the *V. dahliae* colony in the presence of antagonistic bacterium (cm) [6].

This experiment was conducted twice with three biological replicates per each interaction and used medium. Analysis of variance (ANOVA) for PI scores was carried out and mean values were compared using Tukey HSD Test at *p* = 0.05 via Statistix 10 program (NH Analytical Software, Roseville, MN, USA).

### 4.7. Biocontrol Effectiveness of Pseudomonas simiae PICF7 Mutants against Verticillium Wilt of Olive

Two independent experiments were carried out in order to assess the biocontrol performance of the six selected PICF7 mutants (Table 4) against the D pathotype of *V. dahliae* under non-gnotobiotic conditions. Non-inoculated (control), *V. dahliae*-inoculated (disease control), PICF7 wild-type/*V. dahliae*-inoculated and PICF7 mutants/*V. dahliae*-inoculated plants were the 9 different treatments (12 plants per treatment) included in the bioassays. Nursery-produced olive plants cv. Picual (5-month old) qualified as very susceptible to the D pathotype [93] were used. Before starting the bioassays, plants were acclimated in a greenhouse under natural lighting and a temperature range of 21–26 °C for one month. One day before the inoculation with bacteria, olive plants were carefully uprooted and transplanted into polypropylene pots (11 × 11 × 12 cm, one plant per pot) containing the same potting substrate used in the nursery.

Inocula of *P. simiae* PICF7 wild-type and selected mutants were grown in LB agar plates at 28 °C for 48 h (Tc selective pressure was kept for the mutants). Bacterial cells were scraped from the medium with 5 mL of 10 mM MgSO_4_7H_2_O. Plants were inoculated by drenching with 150 mL per pot of each bacterial cell suspension (1 × 10^8^ cfu/mL), prepared as described by Gómez-Lama Cabanás and coworkers [6], or 10 mM MgSO_4_.7H_2_O for the disease control and control treatments (see above). One week after bacterial inoculation, plants were challenged with the pathogen following the method earlier described [6]. Non-inoculated (control) plants were watered with just 150 mL of water. Plants were randomly distributed in three blocks (each containing 4 plants per treatment).

Disease symptoms (defoliation, chlorosis and wilting) were assessed twice a week during 100 days after pathogen inoculation. To evaluate the disease progress, a scale from 0 to 4 was used according to the percentage of affected leaves and twigs (0, healthy plant; 1, 1–33%; 2, 34–66%; 3, 67–100%; and 4, dead plant). The estimated AUDPC [94] was calculated based on data collected over time. Parameters such as DI, M, FS and DII were also calculated for each treatment. Final DI and M were established as the percentage of affected or dead plants, respectively, at the end of each independent bioassay. DII was calculated as:

$$\mathrm{DII}\;=\;\frac{\Sigma Si\;\times \;Ni}{4\;\times \;Nt}$$

where *Si* is the severity of symptoms, *Ni* is the number of plants with *Si* symptoms severity, and *Nt* is the total number of plants. Analysis of variance (ANOVA) for AUDPC and FS parameters was performed using the Statistix 10 software (Analytical Software, Tallahassee, FL, USA). Significant differences among means were compared using the Fisher’s Least Significant Difference (LSD) test (*p* < 0.05).

## 5. Conclusions

Genes of *P. simiae* PICF7 involved in copper tolerance (i.e., putatively coding for the transcriptional regulator CusR and the chemotaxis protein CheV) and biofilm production (i.e., encoding a membrane protein and a flagellar regulatory protein (*fleQ*)) have been identified. Mutant analysis has demonstrated that these phenotypes, as well as phytase activity, are not involved in the effective control of VWO exerted by this rhizobacteria. Nevertheless, the colonization pattern of biofilm-defective mutants was altered compared to that of the wild-type strain. Indeed, inner colonization of olive roots was never observed for these mutants, indicating that biofilm formation is needed to fully develop an endophytic lifestyle. While *P. simiae* PICF7 offers interesting agro-biotechnological perspectives as for its copper-tolerance, biocontrol effectiveness and endophytic lifestyle, among other beneficial traits, further research will be necessary to elucidate the actual mechanisms explaining control of VWO by this BCA.

## Figures and Tables

**Table 1:** Disrupted genes identified in the selected *Pseudomonas simiae* PICF7 mutants.

Mutant Name	Length of the DNA Sequence Analyzed	Phenotype	Accession Number	Closest Strain	Query Cover (%)	E-Value	Identity (%)	Definition (GenBank)	Definition (GenName)
**Cop1**	294	Modified copper tolerance	WP_069672392.1	*Pseudomonas simiae* WCS417	61	1 × 10^−31^	100	chemotaxis protein CheV	*cheV*
**Cop33**	549		WP_176332083.1	*Pseudomonas simiae* WCS417	41	8 × 10^−44^	100	Heavy metal response regulator transcription factor	*cusR*
**Bfm8**	468	Impaired in biofilm formation	PHX42735.1	*Pseudomonas simiae* PCL1751	100	7 × 10^−108^	100	ATPase AAA	*fleQ*
**Bfm9**	212		WP_045791339.1	*Pseudomonas simiae* PCL1751	99	4 × 10^−21^	100	membrane protein	- ^1^

Identification of the disrupted genes by Tn5-Tc^R^ transposon insertion was carried out using a combination of arbitrary and nested-PCR strategy, followed by sequence comparison using the BLASTn and BLASTx algorithms available at the NCBI website. ^1^ No definition provided.

**Table 2:** Percentage of growth inhibition exerted over *Verticillium dahliae* V937I (defoliating pathotype) by wild-type PICF7 and mutants.

	Experiment I			Experiment II	
	Media			Media	
Strain	PDA	NA	Strain	PDA	NA
PICF7	41.49	21.70	PICF7	47.69	21.90
Cop1	47.68	27.63	Cop1	42.38	26.97
Cop33	41.72	26.97	Cop33	42.38	23.68
Bfm8	64.36 *	29.72 *	Bfm8	46.15	21.90
Bfm9	37.77	20.75	Bfm9	54.36 *	25.24
Phy17	ni	25.47	Phy17	0.51 *	30.48
Phy18	ni	21.23	Phy18	1.03 *	18.10

Asterisks mean significant difference compared to the wild-type strain (*p* ≤ 0.05) according to Tukey HSD Test. At least three biological replicates for each dual confrontation and culturing medium were performed. PICF7, *Pseudomonas simiae* PICF7; PDA, Potato Dextrose Agar; NA, Nutrient Agar; ni, no inhibition observed.

**Table 3:** Assessment of biocontrol activity of *Pseudomonas simiae* PICF7 and selected mutants against *Verticillium dahliae* V937I (defoliating pathotype).

	Experiment I					Experiment II				
	Disease Parameters					Disease Parameters				
Treatments	FS	Final DI (%)	Final DII	M (%)	AUDPC	FS	Final DI (%)	Final DII	M (%)	AUDPC
*V. dahliae*	2.13a	91.67	0.53	25	88.11a	2.79a	91.67	0.70	41.67	101.64a
*V. dahliae*/PICF7	1.17b	75	0.29	16.67	31.43b	2.04ab	66.67	0.51	33.33	83.75ab
*V. dahliae*/Bfm8	0.63b	91.67	0.15	0	19.71b	1.58bc	66.67	0.40	16.67	59.00bc
*V. dahliae*/Bfm9	1.00b	75	0.25	0	29.24b	1.50bc	50	0.38	33.33	56.10bc
*V. dahliae*/Phy17	0.81b	83.33	0.2	0	19.02b	0.96c	50	0.24	8.33	38.19c
*V. dahliae*/Phy18	0.85b	75	0.21	8.33	24.35b	1.04c	33.33	0.26	16.67	30.06c
*V. dahliae*/Cop1	1.27b	83.33	0.31	8.33	40.09b	1.56bc	66.67	0.39	25	49.58bc
*V. dahliae*/Cop33	0.92b	91.67	0.22	0	31.51b	1.31bc	66.67	0.33	16.67	43.44bc

FS, mean (*n* = 12) of disease severity symptoms (from 0 to 4) scored at the end of the experiments. Final DI, final disease incidence (%). Final DII, final disease intensity index (ranging 0–1) calculated with data on incidence and severity of symptoms. M, dead plants at the end of the experiments (%). AUDPC, area under the disease progress curve. Data are the average of three randomly distributed blocks each with four pots (plants) per treatment. In each column, values followed by different letters are significantly different according to Fisher’s protected LSD test (*p* < 0.05). All disease parameters were calculated at the end of the experiments (100 days).

**Table 4:** Bacterial strains, fungal pathogen and plasmid used in this study.

Microorganisms and Plasmid	Characteristics	Reference/Source
**Bacterial Strains and Mutants**		
*Escherichia coli* DH5α	*recA1 endA1* Φ80d *lacZ dam-15*	Clontech
*Bacillus* sp. PIC28	PGPR, negative control used in phytase activity assays	[7]
*Pseudomonas indica* PIC128	PGPR, negative control used in biofilm formation assays, isolated from the rhizosphere of a nursery-produced olive plant	Lab. Plant-Microorganism Interactions/This study
*Pseudomonas putida* KT2440	PGPR, positive control used in biofilm formation assays	[84]
*Pseudomonas simiae* PICF7	Wild-type PGPR	[14]
Cop1	PICF7 Tn5-Tc^R^ mutant (*cheV*::Tn*5*-Tc^R^) affected in copper resistance	This work
Cop33	PICF7 Tn5-Tc^R^ mutant (*CusR*::Tn*5*-Tc^R^) affected in copper resistance	This work
Bfm8	PICF7 Tn5-Tc^R^ mutant (*fleQ*::Tn*5*-Tc^R^) impaired in biofilm formation	This work
Bfm9	PICF7 Tn5-Tc^R^ mutant impaired in biofilm formation	This work
Phy17	PICF7 Tn5-Tc^R^ mutant deficient in phytase activity	This work
Phy18	PICF7 Tn5-Tc^R^ mutant deficient in phytase activity	This work
*P. simiae* PICF7 (pLRM1)	PICF7 (Gm^R^) GFP-labeled mutant derivative	[19]
Cop1 (pLRM1)	Cop1 (Tc^R^ and Gm^R^) GFP-labeled mutant derivative	This work
Cop33 (pLRM1)	Cop33 (Tc^R^ and Gm^R^) GFP-labeled mutant derivative	This work
Bfm8 (pLRM1)	Bfm8 (Tc^R^ and Gm^R^) GFP-labeled mutant derivative	This work
Bfm9 (pLRM1)	Bfm9 (Tc^R^ and Gm^R^) GFP-labeled mutant derivative	This work
Phy17 (pLRM1)	Phy17 (Tc^R^ and Gm^R^) GFP-labeled mutant derivative	This work
Phy18 (pLRM1)	Phy18 (Tc^R^ and Gm^R^) GFP-labeled mutant derivative	This work
**Plasmid**		
pLRM1	pBBR1-MCS5 carrying a fusion of the P_A1/04/03_ promoter to the gfpmut3* gene, Gm^R^	[85]
**Fungal pathogen**		
*Verticillium dahliae* V937I	Representative of the defoliating pathotype, originating from a diseased olive tree	[86]

Tc, tetracycline; Gm, gentamicin; GFP, Green Fluorescence Protein, PGPR, Plant Growth Promoting Rhizobacteria.

## Data Availability

All data required to reproduce the results presented in this study can be found in the article.
